# Animal welfare assessment protocol for quails reared for meat production

**DOI:** 10.3389/fvets.2024.1452109

**Published:** 2024-09-30

**Authors:** Antoni Dalmau, Lorena Padilla, Aranzazu Varvaró-Porter, Aida Xercavins, Antonio Velarde, Alexandra Contreras-Jodar

**Affiliations:** Animal Welfare Program, IRTA, Monells, Girona, Spain

**Keywords:** animal-based protocol welfare, assessment protocol, behaviour, certification, feeding, health, housing, outputs

## Abstract

**Introduction:**

It is estimated that 1.4 billion quails are reared each year for their eggs and meat, but animal welfare assessment protocols for this species have yet to be established. The objective of this study was to devise an animal welfare assessment protocol developed through a multidimensional approach that contained a number of animal-based indicators (ABIs) for quails (*Coturnix japonica*) reared for meat production.

**Methods:**

During 2021 and 2022, the identical auditor visited and audited 14 Spanish farms in their initial year of integration into an animal welfare certification scheme. The protocol is categorised into 4 principles and 12 criteria. The “good feeding” principle includes 6 indicators (1 ABI), “good housing” includes 10 indicators (5 ABIs), “good health” includes 12 indicators (9 ABIs), and “appropriate behaviour” contains 8 indicators (5 ABIs). The final welfare assessment is calculated at the farm level using scores from the on-farm recordings. The assessment is a step-by-step weighted sum of the scores from the various indicators, with the final score ranging between 0 and 100.

**Results and discussion:**

The main welfare issues found on all farms were a lack of temperature and humidity records, a poor lighting pattern, and the absence of an outdoor range or access to one. To a lesser degree, it was also found that there were excessive numbers of birds per feeder, the presence of improperly functioning drinkers (i.e., not working, inadequate water flow, or dripping water), poor litter quality, and a high prevalence of birds with dirty plumage and lameness. Despite this, the farms achieved a good overall score, being classified as “enhanced” (*n* = 11) and “acceptable” (*n* = 3). The tool proved helpful in identifying specific welfare issues at the farm level and conducting benchmarking.

## 1 Introduction

The most commonly used species of quail for production is the Japanese quail (*Coturnix japonica*). Commercial genetic selection for quail has mainly focused on increasing body weight and egg production rate (1, 2). Despite using the same species for both egg and meat production, the genetic line for egg production is lighter (<200 g) than the genetic line for meat production (>300 g). It is estimated that 1.4 billion quails are reared annually for their eggs and meat, but there are no specific numbers on this. For instance, the species cannot be identified on the Statistics page of the Food and Agriculture Organization (FAOSTAT) due to the insignificant numbers it represents (3). According to the numbers found in FAOSTAT, chickens (*Gallus gallus domesticus*) contribute 90% of world meat production, followed by turkeys (*Meleagris gallopavo domesticus*) with 5%, ducks (*Anas platyrhyncos domesticus* and *Cairina moschata domestica*) with 4%, and guinea fowl (*Numida meleagris*) with 0.2%. Quail meat would be included in the remaining 0.8%, which also includes other species, such as geese (*Anser cygnoides*), pigeons (*Columba livia domestica*), ostriches (*Struthio camelus*), and pheasants (*Phasianus colchicus*). Most quails are farmed in China, with a production rate of over 80%. In the European Union (EU), where the production of this species was introduced in the 1950s, it is estimated that over 100 million quails are produced. The domesticated Japanese quail is capable of attaining adult weight within 5–6 weeks of hatching (4) and becoming sexually mature within 35–56 days of age (5). The average age at slaughter is often 4–5 weeks, with body weights ranging from 140 to 300 g (6, 7). Quails used for meat production are usually kept in deep-lit indoor floor systems. The space allocation varies between 89 and 147 cm^2^ per bird, with an average of 113.80 cm^2^. The height of floor systems typically ranges from 2 to 3 metres, enabling the caretakers to enter the pen and the birds to perform brief flights. Group sizes range from 30,713 to 118,721 birds per building (5). Usually, artificial light is provided for a duration of 14 to 16 h per day, and the room temperature ranges from 18°C to 20°C. Feed is usually provided in round troughs, and water is typically provided to nipple drinkers with a range between 30 and 52 quail per drinker (5).

The World Organisation for Animal Health (WOAH) states that a good level of welfare exists when the animal is healthy, comfortable, well-nourished, safe, and capable of expressing its innate behaviour without any suffering, pain, fear, or anguish. In the last few decades, animal welfare has become a growing concern for society. In this regard, the European Commission conducted a study by surveying European citizens, of which 91% considered it important to protect the welfare of farmed birds, 67% stated that they would like to obtain more information about animal production conditions, and 60% mentioned that they were willing to pay more for products (8). In connection with the previous point, the EU provided funding for one of the most ambitious projects ever undertaken on animal welfare, namely the Welfare Quality^®^ project, from 2004 to 2009. This project aimed to develop protocols to assess animal welfare in an objective, scientific, and practical way, with a focus on animal-based measures (9). However, this project was primarily focused on raising cattle, pigs, and chickens. After this project, the EU funded a second one, the European Animal Welfare Indicators (AWIN) project, which covered some of the omitted species from the previous one, including turkeys, sheep, goats, and horses. Yet other species, such as rabbits or quails, were never considered in either of the two European initiatives. Nonetheless, quail (or rabbit) producers face the same challenges as other producers, including a higher demand from consumers for animal-friendly production systems and greater production efficiency to increase marginal benefits. In 2020, two protocols were published for rabbits reared for meat purposes using the Welfare Quality^®^ approach to achieve a better understanding of animal welfare and the tools for its evaluation (10, 11). These tools, commonly known as animal welfare assessment protocols, play a key role as they can be utilised by farmers to identify critical points in their farms for investment, to compare their own results with those from other producers to perform self-assessments, and to establish communication channels with consumers, thereby enhancing the value of their farms through improved conditions.

A welfare assessment describes the welfare of animals at farm and slaughterhouse levels by means of a series of measures (i.e., factors that may be measured or assessed and reflect animal welfare). Welfare assessments include resources provided in the form of housing systems and management routines as well as the manner in which the birds respond, including clinical and behavioural indicators. At the same time, the assessment includes the animal’s positive emotions and experiences instead of simply measuring negative responses. Welfare Quality^®^ assessments under the scope of Welfare Quality^®^ are based on four principles: good feeding, good housing, good health, and appropriate behaviour. Within these principles, 12 specific animal welfare criteria have been defined, each of which includes a number of indicators that prioritise animal-based measures (12). The objective of this study was to present a protocol based on the Welfare Quality^®^ approach developed for quail reared for meat production for discussion subsequent to its implementation in 14 farms assessed in Spain that were interested in achieving certification on animal welfare.

## 2 Methods

**Figure 1:**
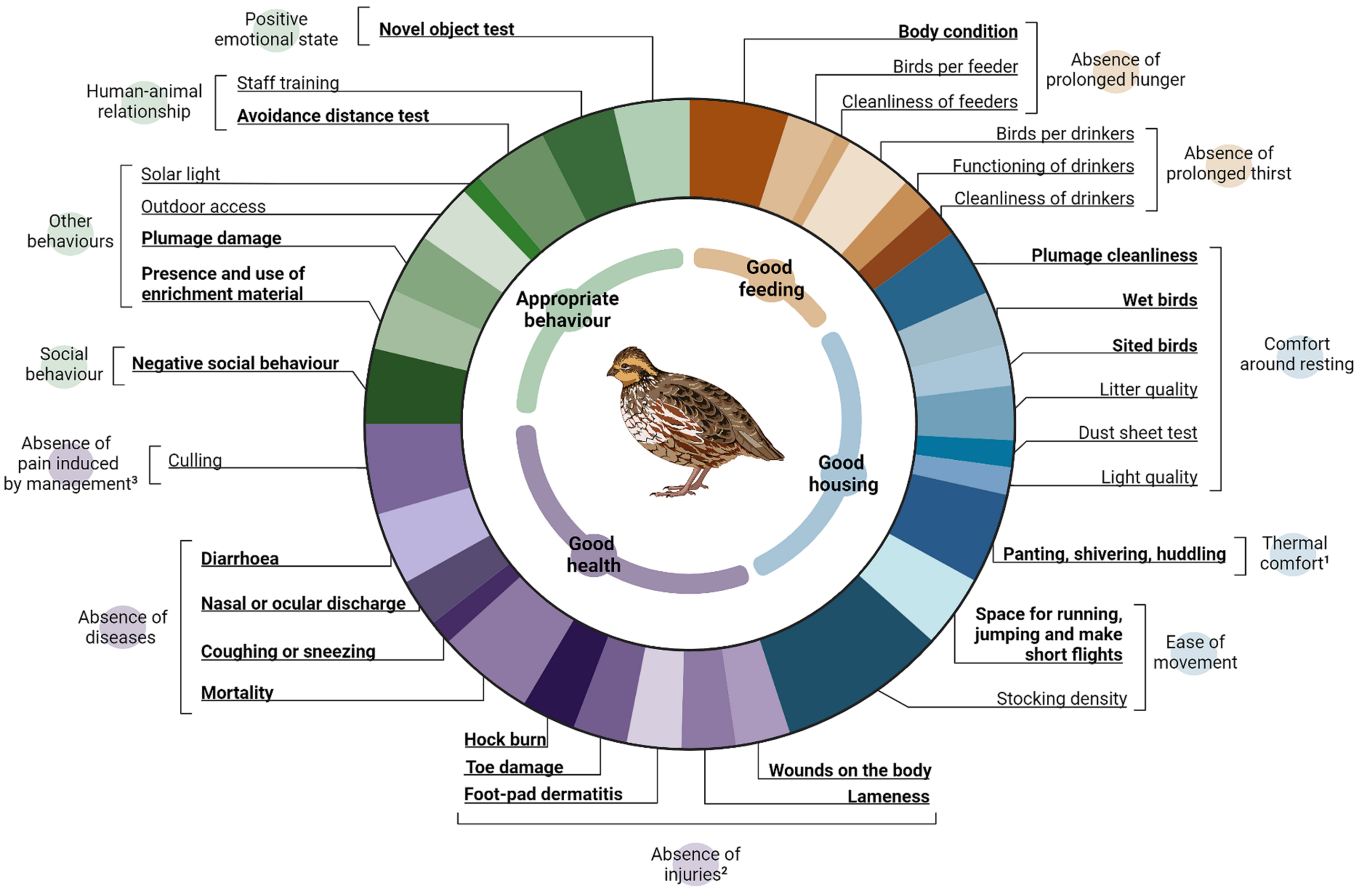
Pie chart displaying the proportion of the principles (*n* = 4), criteria (*n* = 12) and indicators (*n* = 37) used for assessing the meat quail welfare. The arcs in the doughnut hole represent the principles of Good feeding (orange), Good housing (blue), Good health (purple), and Appropriate behaviour (green). Labels protruding from the chart show the indicators used; in bold, those animal-based indicators. At the outer part are the criteria enclosing each of the indicators included. Superscript 1 designates temperature registers that represent the same proportion as the indicator including the thermal comfort criterion, but with a negative score (from 0 to −100). Superscript 2 designates the risk of injuries due to the surroundings indicator, which represents the same proportion as the indicators included in the absence of injuries criterion, but with a negative score (from 0 to −100). Superscript 3 designates an emergency killing indicator that represents the same proportion as the indicator culling but with a negative score (from 0 to −100).

During a single visit from summer 2021 to autumn 2022, 14 Spanish quail farms were assessed using the animal welfare protocol developed within the scope of the study. When the meat quails were at least 26 days old, the assessment was carried out. A sampling method was used to assess some birds in different locations within the farm to ensure that they were representative of the overall picture of the farm. All farms were audited by the same auditor, who was trained according to the Welfare Quality^®^ training procedure (9) and followed the certification requirements. In the present study, the structure provided by the Welfare Quality^®^ scheme was utilised, wherein 12 criteria comprised of 4 distinct principles were evaluated based on 36 specific welfare indicators in quails (Figure 1). These welfare indicators comprised 20 measures based on direct animal observations (animal-based indicators; ABIs) individually sampled in 250 quails from at least 10 different locations within the barn, and 16 measures focussing on housing and facilities (resource-based indicators; RBIs), collected in a group or the facilities, considering 10 different locations within the barn. Thus, “good feeding” includes 6 indicators (1 ABI and 5 RBIs) within two criteria; “good housing” includes 10 indicators (5 ABIs and 5 RBIs) within three criteria; “good health” includes 12 indicators (9 ABIs and 3 RBIs) within three criteria; and “appropriate behaviour” contains 8 indicators (5 ABIs and 3 RBIs) within four criteria. Each of these welfare indicators was assessed on a scale ranging from 0 to 100 points, and wherever feasible, a three-point scale of 0 for good welfare, 1 for compromised welfare, and 2 for poor welfare was employed. The final score could range from 0 to 100 points, and the goal for the farmer was to achieve at least 55 points out of 100.

### 2.1 Good feeding

The Good Feeding Principle was assessed by means of the combination of six indicators included in the criteria: Absence of prolonged hunger (55% of the total score of the Good Feeding Principle) and Absence of prolonged thirst (45% of the total score; Table 1).

**Table 1:** Indicators used to assess the criteria of Absence of prolonged hunger and Absence of prolonged thirst in quails for meat production.

Criterion	Indicator	Weight	Definition of categories	Score
Absence of prolonged hunger	Body condition	60%	0% of lean birds	100
			<0.5% of lean birds	80
			<1.0% of lean birds	60
			<1.3% of lean birds	40
			<2.5% of lean birds	20
			≥2.5% of lean birds	0
	Birds per feeder	30%	≥0.40 cm per animal	100
			≥0.38 cm per animal	80
			≥0.35 cm per animal	60
			≥0.32 cm per animal	40
			≥0.30 cm per animal	20
			<0.30 cm per animal	0
	Cleanliness of feeders	10%	100% of clean feeders (score 0)	100
			90% of clean feeders	80
			80% of clean feeders	60
			70% of clean feeders	40
			50% of clean feeders and/or one very dirty feeder (score 2)	20
			<50% of clean feeders and/or two very dirty feeders (score 2)	0
Absence of prolonged thirst	Birds per drinker	50%	One drinker every 25 birds	100
			One drinker every 30 birds	80
			One drinker every 35 birds	60
			One drinker every 40 birds	40
			One drinker every 45 birds	20
			One drinker for 46 birds or more	0
	Functioning of drinkers	25%	100% nipple drinkers with cups having good water flow	100
			90% nipple drinkers with cups having a good water flow, no leaking drinkers	80
			50% nipple drinkers with cups having a good water flow, no leaking drinkers	60
			<50% nipple drinkers with cups having a good water flow, ≤10% leaking drinkers	40
			<20% leaking drinkers	20
			≥20% leaking drinkers	0
	Cleanliness of drinkers	25%	100% of clean drinkers	100
			90% of clean drinkers	80
			80% of clean drinkers	60
			70% of clean drinkers	40
			60% of clean drinkers	20
			<60% of clean drinkers	0

The weight means which percentage of the score of the total criterion is represented by each parameter. Each indicator is assessed according to different categories and is scored accordingly.

For the evaluation of the criterion “Absence of prolonged hunger”, body condition (ABI represents 60% of the total score of the criterion) was assessed visually and by palpation. When thinness was evident, the keel was prominent due to a lack of muscle around the keel (depressed contour), or when the keel was clearly underweight compared to other birds of the same age, the body condition was assessed. When the percentage of lean birds reaches 2.5%, they score 0 points (Table 1). In contrast, the body condition was considered excellent (100 points) when the percentage of too-lean birds was 0%. The bird per feeder (RBI, 30%) was calculated by dividing the total number of the animals that entered the house by the total length of the feeding troughs or stalls, ranging from ≥0.4 cm per animal (100 points) to <0.30 cm per animal (0 points). According to previous protocols, the cleanliness and condition of feeders (RBI, 10%) were rated on a three-point scale (10, 11): 0 if there was no dirt inside and no risk of injury to the birds; 1 if it was partially dirty, but no risk of injury; and 2 if there was a risk of injury to the birds or the trough was very dirty. A feeder was deemed to be dirty if it contained corrupted food, compacted dry food, and mould. The 100 points were obtained when 100% of the feeders were rated with a 0 on the three-point scale. In contrast, if fewer than 50% of feeders or more were scored with a 0 and/or two or more scored with a 2, then 0 points were obtained.

The Absence of prolonged thirst (45%) was evaluated using three RBIs: drinking points per quail (50%), functioning of drinkers (25%), and cleanliness of drinkers (25%). Birds per drinker were calculated by dividing the total number of birds that entered the house by the total number of drinking points available (12), with one drinker per 25 birds (100 points) and one drinker per 46 or more birds (0 points). Drinkers were assessed on a three-point scale in accordance with previous protocols (10, 11). If the drinker worked properly, had a good flow of water, and there was a cup for recovering water; 1 if the drinker worked properly with a good flow of water, but there was no cup to recover the water that was not drunk by the animal; 2 if the drinker had an insufficient flow, was dripping, or was in any other situation showing a deficient state of conservation. If 100% of the drinkers had a score of 0 and 100 points were awarded, no points were awarded, and if more than 20% of the drinkers had a score of 2, no points were awarded. The cleanliness of the drinkers (10, 11) was assessed in at least 10 different locations of the farm and was scored as 0 (clean) or 2 (dirty). A dirty drinker was considered to be one with the presence of oxide, corrupted food, compacted dry food, or mould. If 100% of the drinkers were rated with a 0, 100 points were obtained; if two or more drinkers were rated with a 2, no points were obtained.

### 2.2 Good housing

The Good Housing Principle was assessed by combining three criteria: comfort around resting (45%), thermal comfort (15%), and ease of movement (40% of the total score; Table 2).

**Table 2:** Indicators used to assess the criteria of comfort around resting, thermal comfort and ease of movement in quails for meat production.

Criterion	Indicator	Weight	Definition of categories	Score
Comfort around resting	Plumage cleanliness	25%	100% of birds with ≤20% of the breast area soiled	100
			<1.0% of birds with 20 to 50% of the breast area soiled, 0% of birds with more than 50% of the breast area soiled	80
			<3.0% of birds with 20 to 50% of the breast area soiled, < 0.5% of birds with more than 50% of the breast area soiled	60
			<5.0% of birds with 20 to 50% of the breast area soiled, < 1.0% of birds with more than 50% of the breast area soiled	40
			<10.0% of birds with 20 to 50% of the breast area soiled, < 1.5% of birds with more than 50% of the breast area soiled	20
			≥10.0% of birds with 20 to 50% of the breast area soiled, ≥ 1.5% of birds with more than 50% of the breast area soiled	0
	Wet birds	20%	0% of wet birds	100
			<0.5% of wet birds	80
			<1.0% of wet birds	60
			<1.5% of wet birds	40
			<2.0% of wet birds	20
			≥2.0% of wet birds	0
	Sited birds	15%	≥50% of sited birds	100
			≥40% of sited birds	80
			≥30% of sited birds	60
			≥20% of sited birds	40
			≥10% of sited birds	20
			<10% of sited birds	0
	Litter quality	20%	100% of the assessed points scored with a 0	100
			100% of the assessed points scored with a 0 or with a score of 1	80
			Up to one point assessed with a score of 2	60
			Up to three points scored with 2 and one point scored with 3	40
			More than three points scored with a 2, up to three points scored with 3 and up to one point scored with 4	20
			Any other case	0
	Dust sheet test	10%	No dust presence in any area	100
			Only one location with a score of 1	80
			Two or more locations with a score of 1	60
			Only one location with a score of 2	40
			Up to two locations with a score of 2	20
			More than two locations with a score of 2	0
	Light quality	10%	All the locations were assessed with a score of 0	100
			One location with difficulties in seeing the birds	60
			Two locations with difficulties in seeing the birds	20
			More than two locations with difficulties in seeing the birds or less than 8 h of light or darkness per day	0
Thermal comfort	Panting/shivering/huddling	100%	0% of birds panting, shivering or huddling	100
			<10% of birds panting, shivering or huddling	80
			<20% of birds panting, shivering or huddling	60
			<30% of birds panting, shivering or huddling	40
			<40% of birds panting, shivering or huddling	20
			≥40% of birds panting, shivering or huddling	0
	Temperature registers	−100%	THI < 32 and Temp never below 5°C	0
			THI < 38 and Temp never below 1°C	−45
			No registers or any other case	−100
Ease of movement	Space for running, jumping and making short flights	30%	100% of the birds are able to perform these behaviours	100
			At least 50% of the birds are able to perform these behaviours	55
			Less than 50% of the birds are able to perform these behaviours, but at least some are able	20
			No birds present are able to perform these behaviours	0
	Stocking density	70%	At least 140 cm^2^ per quail	100
			At least 125 cm^2^ per quail	80
			At least 115 cm^2^ per quail	60
			At least 100 cm^2^ per quail	40
			At least 90 cm^2^ per quail	20
			<90 cm^2^ per quail	0

The weight means which percentage of the score of the total criterion is represented by each indicator. Each indicator is assessed according to different categories and is scored accordingly. THI, Temperature and Humidity Index.

The comfort around resting criteria was assessed through plumage cleanliness (ABI, 25%), wet birds (ABI, 20%), sited birds (ABI, 15%), litter quality (RBI, 20%), dust sheet test (RBI, 10%), and light pattern and quality (RBI, 10%). Plumage cleanliness was assessed in a total of 250 quails selected in at least 10 different locations within the farm. It was assessed on the ventral part of the animal and the breast, not considering the cloaca. It was rated on a three-point scale according to previous protocols (10–12, and): 0 if less than 20% of the surface was dirty; 1 if 20–50% of the surface was dirty; and 2 if >50% of the surface was dirty. The score ranged from 100 points when 100% of the birds received a 0 rating to 0 points when 10% or more received a 1 rating or when 1.5% or more received a score of 2 (Table 2). Wet birds were assessed in 250 quails selected from at least 10 different locations within the farm. It was considered a wet animal if any part of the body (except for the mucosas) contained water, even if there was a very small drop of water on the head (10). These scores ranged from 0% of wet birds (100 points) to 2.0% or more of wet birds (0 points; Table 2). The birds that were located were assessed by observing and ensuring that they did not disturb a group of birds. This assessment should be conducted at least three times during the audit: at the beginning (using the same groups utilised for the assessment of social behaviour and the presence of coughing/sneezing), at half the audit (after lameness, while thermoregulation is assessed), and at the end (while thermoregulation is assessed again) and in a minimum of 100 birds each time. A sited quail was considered a quail resting or sleeping (sited or lying down) on a horizontal surface, and the score ranged from 50% of the birds sited (100 points) to <10% of the birds sited (0 points; Table 2). The litter quality was assessed at 10 different locations within the farm, and it was rated on a five-point scale (as outlined in the Welfare Quality^®^ Protocol for Broilers; (12)): 0: completely dry and flaky, i.e., easily moves with the foot; 1: dry but not easy to move with the foot; 2: leaves an imprint of the foot and will form a ball if compacted, but the ball does not stay together well; 3: sticks to boots and sticks readily in a ball if compacted; and 4: sticks to boots once the cap or compacted crust is broken. The score ranged from 100 points when all areas had a score of 0 to 0 points if more than one location had a score of 4 (Table 2). The dust parameter was evaluated by means of a black surface measuring approximately 10 × 15 cm (DIN A6) that was left during the assessment at the centre (or entrance) of the building housing the quails, at the same height as their heads, and at least in three different locations of the farm (as outlined in the Welfare Quality^®^ Protocol for Broilers (12)). At the end of the visit, the level of dust accumulation was assessed based on three possibilities: score 0 indicates no evidence of dust, score 1 indicates minimal dust evidence (a thin covering of dust), and score 2 indicates significant dust evidence (possible to write on the paper with a finger or the paper is not visible). The score ranged from 100 points if all zones scored with a zero to 0 points if more than two zones scored with a score of 2 (Table 2). The quality of the light was deemed correct when it was feasible to verify all the birds and when a minimum of 8 h of light and darkness were provided (10, 11). For its evaluation, all the zones of the farm where the birds were observed were considered. The score ranged from 100 points if all the areas were provided with the appropriate lighting and a suitable pattern to 0 points if there were more than two zones with inadequate lighting conditions or if the birds were not provided with 8 h of light and darkness every 24 h (Table 2).

The thermal comfort criterion was assessed through one ABI and one RBI, which included bird panting, shivering, and huddling (100%) and temperature and humidity recordings (−100%). During the audit, birds were assessed for panting, shivering, and huddling in a minimum of 10 groups, and at least three times during the audit with a minimum of 100 birds each time. The first time was when social behaviour and coughing/sneezing were evaluated; the second time was 1 h later; and the third time was at the end of the audit. Panting was defined as breathing rapidly in short gasps. Shivering is a slow and irregular vibration of any body part or the entire body. Huddling was considered when quails cluster together in tightly packed groups, sitting closely alongside each other, often in “clumps,” with a small amount of empty space in between (according to the Welfare Quality^®^ criteria definitions; (12)). Huddling was distinguished from the usual “loose grouping” that quails display when resting. The scores ranged from 0% of the birds displaying any of the three thermoregulation indicators (100 points) to 40% or more of the birds showing any of the three indicators (0 points; Table 2). The temperature parameter was assessed based on the temperature and humidity data record in the farm (10) taking into account that the temperatures can vary dramatically from the moment the birds enter the farm to the moment they are close to the slaughter weigh. If there were no data, a score of 2 was given. If there were any data, only those from the last 15 days before slaughtering the birds were considered. When the combination of temperature and humidity yielded a value lower than 32 and when the minimum temperature did not fall below 5°C, a score of 0 was assigned. A score of 1 was awarded when the combination of temperature and humidity yielded a range of 32 to 38 and the minimum temperature did not fall below 1°C. If the combination of temperature and humidity reaches a Temperature and Humidity Index (THI) of 39 or the minimum temperature falls below 1°C, the score will be 2. This parameter was applied to rectify the data obtained through the ABIs. Consequently, a score of 0 was not summed, and the score obtained with panting, shivering, or huddling was respected. In the case of a score of 1, 45 points were rested on the score obtained for panting/shivering/huddling, and in the case of a score of 2, the score for all the criteria of thermal comfort was 0 points, regardless of the score obtained for the indicator panting/shivering/huddling.

The ease of movement criterion was assessed through one ABI and one RBI, being: space for running, jumping, and making short flights (30%) and stocking density (70%). After the assessment of all the parameters of the protocols, the parameter space for running, jumping, and making short flights was assessed as a general impression of the whole farm. It does not ask for a specific number of birds or locations, and it was based on a measure (horizontal and vertical movement) used in the Welfare Quality^®^ Laying Hens Protocol (12). The score is considered 0 when the birds were able to run, jump and make short flights without risk of being damaged and 2 if, due to obstacles or any other circumstances, birds could not perform these behaviours. One hundred points were awarded if 100% of the birds had the possibility of performing this behaviour if they wanted, and 0 points were awarded if 100% of the birds were not able to do it (Table 2). The stocking density (10–12) was assessed in the whole facility where the birds were housed, and the verandas or other external areas were considered only when 24 h of permanent access were ensured. The calculation was based on the total space available for the birds to move divided by the number of birds on day 0, at placement, not at the moment of the audit. The score varied from 140 cm^2^ per animal (100 points) to <90 cm^2^ per animal (0 points; Table 2).

### 2.3 Good health

The Good Health Principle was assessed by means of the combination of three criteria: absence of injuries (45%), Absence of diseases (40%), and Absence of pain induced by management (15%, Table 3).

**Table 3:** Indicators used to assess the criteria of absence of injuries, Absence of diseases, and Absence of pain induced by management in quails for meat production.

Criterion	Indicator	Weight	Definition of categories	Score
Absence of injuries	Wounds on the body	20%	0% of birds with a score of 1 or a score of 2	100
			<0.5% of birds with a score of 1	80
			<1.0% of birds with a score of 1 and/or <0.5 of birds with a score of 2	60
			<1.5% of birds with a score of 1 and/or <1.0 of birds with a score of 2	40
			<3.0% of birds with a score of 1 and/or <2.0 of birds with a score of 2	20
			≥3.0% of birds with a score of 1 and/or ≥2.0 of birds with a score of 2	0
	Lameness	20%	<1.0% of birds with a score of 1 and 0% of birds with a score of 2	100
			<2.0% of birds with a score of 1 and/or <0.5 of birds with a score of 2	80
			<3.0% of birds with a score of 1 and/or <1.0 of birds with a score of 2	60
			<5.0% of birds with a score of 1 and/or <1.5 of birds with a score of 2	40
			<7.0% of birds with a score of 1 and/or <3.0 of birds with a score of 2	20
			≥7.0% of birds with a score of 1 and/or ≥3.0 of birds with a score of 2	0
	Food pad dermatitis	20%	<2.0% of birds with a score of 1 and/or <1% of birds with a score of 2	100
			<4.0% of birds with a score of 1 and/or <2.0 of birds with a score of 2	80
			<8.0% of birds with a score of 1 and/or <4.0 of birds with a score of 2	60
			<12.0% of birds with a score of 1 and/or <6.0 of birds with a score of 2	40
			<15.0% of birds with a score of 1 and/or <8.0 of birds with a score of 2	20
			≥15.0% of birds with a score of 1 and/or ≥8.0 of birds with a score of 2	0
	Toe damage	20%	<5% of birds with the toe damage	100
			<8% of birds with the toe damage	80
			<10% of birds with the toe damage	60
			<15% of birds with the toe damage	40
			<20% of birds with the toe damage	20
			≥20% of birds with the toe damage	0
	Hock burn	20%	<0.5% of birds with a score of 1 and 0% of birds with a score of 2	100
			<1.0% of birds with a score of 1 and/or <0.5 of birds with a score of 2	80
			<2.0% of birds with a score of 1 and/or <1.0 of birds with a score of 2	60
			<4.0% of birds with a score of 1 and/or <2.0 of birds with a score of 2	40
			<8.0% of birds with a score of 1 and/or <4.0 of birds with a score of 2	20
			≥8.0% of birds with a score of 1 and/or ≥4.0 of birds with a score of 2	0
	Risk of injuries due to the surroundings	−100%	No elements with risk of injuries	0
			1 element with risk of injuries	−10
			2 elements with risk of injuries	−20
			3 elements with risk of injuries	−30
			4 elements with risk of injuries	−40
			5 elements with risk of injuries	−50
			6 elements with risk of injuries	−60
			7 elements with risk of injuries	−70
			8 elements with risk of injuries	−80
			9 elements with risk of injuries	−90
			10 elements with risk of injuries	−100
Absence of diseases	Mortality	40%	<2% for the whole cycle and <1% for the first week	100
			<3% for the whole cycle and <1.5% for the first week	80
			<4% for the whole cycle and <2% for the first week	60
			<6% for the whole cycle and <3% for the first week	40
			<10% for the whole cycle and <5% for the first week	20
			Any other situation	0
	Coughing or sneezing	10%	No points were assessed with a score of 1 or 2	100
			One point was assessed with a score of 1	80
			Two points were assessed with a score of 1 and one with a score of 2	60
			Three points were assessed with a score of 1 and two with a score of 2	40
			Up to five points were assessed with a score of 1 and three with a score of 2	20
			Any other situation	0
	Nasal or ocular discharge	20%	0% of birds with nasal ocular discharge	100
			<0.5% of birds with nasal ocular discharge	80
			<1.0% of birds with nasal ocular discharge	60
			<2.0% of birds with nasal ocular discharge	40
			<5.0% of birds with nasal ocular discharge	20
			≥5.0% of birds with nasal ocular discharge	0
	Diarrhoea	30%	0% of birds with diarrhoea	100
			<0.5% of birds with diarrhoea	80
			<1.0% of birds with diarrhoea	60
			<2.0% of birds with diarrhoea	40
			<5.0% of birds with diarrhoea	20
			≥5.0% of birds with diarrhoea	0
Absence of pain induced by management	Culling	100%	≥70% of the total dead birds being culled	100
			≥60% of the total dead birds being culled	80
			≥50% of the total dead birds being culled	60
			≥40% of the total dead birds being culled	40
			≥30% of the total dead birds being culled	20
			Any other situation, including the absence of registers	0
	Emergency killing	−100%	Good protocol and well-applied	0
			The protocol is not correct, but the killing system is well-applied	−55
			Any other situation	−100

The weight means which percentage of the score of the total criterion is represented by each indicator. Each indicator is assessed according to different categories and is scored accordingly.

The absence of injuries criterion was assessed through the evaluation of five ABI and one RBI, being: wounds on the body (20%), lameness (20%), foot pad dermatitis (20%), toe damage (20%), hock burn (20%), and risk of injuries due to the surroundings (−100%). All the ABIs of this criterion were assessed in a total of 250 quails that were collected from a minimum of 10 distinct locations within the farm, with 25 animals per point. For wounds on the body (10), a lesion was considered a fresh scratch or an open lesion larger than 0.5 cm in any part of the animal that was not healed. A score of 0 was awarded when there were no injuries more than 0.5 cm in any part of the birds, a score of 1 when there was fewer than one lesion exceeding 0.5 cm, and a score of 2 when there was more than one lesion exceeding 0.5 cm or any with more than 1.5 cm. The score ranged from 100 points when no birds with a score of 1 or 2 were found to 0 points when at least 3% of birds were found with a score of 1 or at least 2% with a score of 2 (Table 3). The score of lameness in the present protocol for quails was based on the score for lameness in the Welfare Quality^®^ Protocol for Broilers (12), where 6 categories are considered: (0) Normal, dextrous and agile; (1) Slight abnormality, but difficult to define; (2) Definitive and identifiable abnormality; (3) Obvious abnormality, affects ability to move; (4) Severe abnormality, only takes a few steps; and (5) Incapable of walking. However, for quails only three scores were considered: score 0, no problems, if the animal did not have any difficulty in moving; score 1, moderate problem, if the animal had any difficulty in moving (includes categories 1, 2 and 3 of broilers); and score 2, severe problem, if the animal had several difficulties (no use of one leg or minimum weight bearing; includes scores 4 and 5 for broilers). The score ranged from less than 1% of the observed birds with a score of 1 and none with a score of 2 (100 points) to 7% of birds with a score of 1 or 3% of birds with a score of 2 (0 points; Table 4). For Footpad dermatitis (12), three cases were considered: score 1, no problem, when the feet are fine; score 1, moderate problem, very small lesions or small areas of epithelial proliferation; and score 2, big lesions or epithelial proliferations, signs of inflammation or ulcers. The score varied from less than 2% with a score of 1 and less than 1% with a score of 2 (100 points) to 15% with a score of 1 or 8% with a score of 2 (0 points; Table 3). For toe damage (11) there were considered two possibilities: score 0, no lesions in the toes; score 2, presence of inflammation, balls of dirt, dermatitis, fresh open lesions (with blood or purulent), lost toes or broken toes. The score ranged from less than 5% of the birds with a score of 2 (100 points) to 20% of the birds with a score of 2 (0 points; Table 3). The evaluation of Hock burn (12) was conducted in the area of the tarsus (both legs) and consists of three scores: score 0, indicating no presence of dermatitis or lesion; score 1, indicating the presence of dermatitis; and score 2, indicating the presence of swelling and other signs of inflammation. The score varied from less than 0.5% of the birds with a score of 1 and none with a score of 2 (100 points) to 8% with a score of 1 or 4% with a score of 2 (0 points, Table 3). The last indicator of this criterion was based on the facilities and is entitled Risk of injuries due to the surroundings (10, 11). This was assessed throughout the audit in all the areas where birds were observed and every single thing on the farm that could produce damage to the birds was considered. In this way, every element potentially damaging to the animal was adding 10 points to the whole score of the criterion. In consequence, if the assessor found 10 elements that were dangerous for the birds, 100 points were rested on the total score for the absence of injuries criterion, with independence of the score obtained in the animal-based measures being a total of 0 points (Table 3).

**Table 4:** Indicators used to assess the criteria of social behaviour, other behaviours, human–animal relationship and positive emotional state in quails for meat production.

Criterion	Indicator	Weight	Definition of categories	Score
Social behaviour	Negative social behaviour	100%	No groups were observed with negative social behaviour	100
			One group was observed with negative social behaviour	80
			Two groups were observed with negative social behaviour	60
			Three groups were observed with negative social behaviour	40
			Up to five groups were observed with negative social behaviour	20
			More than five groups were observed with negative social behaviour	0
Other behaviours	Presence and use of the enrichment material, including birds digging and interacting with the litter and grooming	30%	More than 10% of the birds observed doing the described behaviours	100
			Up to 10% of the birds observed doing the described behaviours	60
			Up to 10 birds were observed doing the described behaviours	30
			Any other situation, including the absence of enrichment material	0
	Plumage damage	30%	<0.5% of birds with a score of 1 and 0% of birds with a score of 2	100
			<2.0% of birds with a score of 1 and/or <0.5% of birds with a score of 2	80
			<5.0% of birds with a score of 1 and/or <1.5% of birds with a score of 2	60
			<10.0% of birds with a score of 1 and/or <5.0% of birds with a score of 2	40
			<30.0% of birds with a score of 1 and/or <15.0% of birds with a score of 2	20
			≥30.0% of birds with a score of 1 and/or ≥15.0% of birds with a score of 2	0
	Outdoor access	30%	More than 80% of the birds can stay at the same time in an outdoor area and this is covered at minimum in 40% of the surface	100
			More than 80% of the birds can stay at the same time in an outdoor area, and this is covered at least in 20% of the surface	55
			There is a winter garden (veranda-covered)	40
			Less than 80% of the birds can stay in an area outdoors at the same time, and/or it is covered by less than 20% of the surface	0
	Solar light	10%	All the birds have access to sunlight	100
			At least 50% of the birds have access to sunlight	55
			<50% of the birds have access to sunlight	0
Human–animal relationship	Avoidance distance test	50%	<10% of birds with a score of 1 and <10% of birds with a score of 2	100
			<20% of birds with a score of 1 and/or <20% of birds with a score of 2	80
			<30% of birds with a score of 1 and/or <30% of birds with a score of 2	60
			<50% of birds with a score of 1 and/or <50% of birds with a score of 2	40
			<70% of birds with a score of 1 and/or <70% of birds with a score of 2	20
			≥70% of birds with a score of 1 and/or ≥70% of birds with a score of 2	0
	Staff training	50%	All the staff in contact with birds is trained on animal welfare	100
			At least one person in contact with the birds is trained on animal welfare	55
			None of the persons in contact with the animal is trained in animal welfare	0
Positive emotional state	Novel object test	100%	Five points of observation with a score of 0 and up to two points with a score of 2	100
			Three points of observation with a score of 0 and up to two points with a score of 2	80
			One point of observation with a score of 0 and up to four points with a score of 2	60
			Up to five points of observation with a score of 2	40
			Up to eight points of observation with a score of 2	20
			Any other situation	0

The weight means which percentage of the score of the total criterion is represented by each indicator. Each indicator is assessed according to different categories and is scored accordingly.

The absence of disease criteria was assessed through four ABIs, being mortality (40%), coughing or sneezing (10%), nasal or ocular discharge (20%), and diarrhoea (30%). Mortality (10–12) was defined as the uncontrolled demise of an animal (euthanasia and culling were not taken into account). The mortality rate was arrived at by calculating the average value of the accumulated mortality for each batch housed in the facilities analysed in the recent 12 months. In addition, a second value was taken considering only the first 7 days after entering the facilities for all of these batches. In this manner, the two values were incorporated into the score in such a manner that 100 points were awarded if the total average mortality was less than 2% and the first 7 days were less than 1%. Zero points were awarded when the total average mortality exceeded 10% of the mortality in the first 7 days exceeded 5% (Table 3). The indicator of coughing or sneezing (10) was assessed in 10 different groups of birds for a time of 2 min per group. This was assessed at the same time as social behaviour, sited birds, and thermoregulation. It was rated on a three-point scale: score 0, no birds coughing or sneezing; score 1, no more than two events per point of observation (an event could be a cough or a sneeze); score 2, more than two events per point of observation during the 2 min. The score ranged from 0 points of observation with a score of 1 or 2 (100 points) to more than 5 points with a score of 1 or more than three with a score of 2 (Table 3). The presence or absence of nasal and ocular discharge (10) was only considered. Signs of conjunctivitis were considered as the presence of ocular discharge and liquid in the nostrils as the presence of nasal discharge. The score varied from 0% of birds with ocular or nasal discharges (100 points) to 5% or more with discharges (0 points; Table 3). Diarrhoea (10, 11) was assessed as the presence of liquid faeces around the cloaca of the animal. The score ranged from 0% of birds affected (100 points) to 5% of birds with diarrhoea (0 points; Table 3).

The absence of pain induced by the management criterion was assessed through two RBIs: culling (100%) and Emergency killing methods (−100%). The practice of culling (10) was considered any animal in the farm that, from the moment of placement until the final day of production, was killed by the farmers for productive reasons, health issues, or to prevent any kind of suffering to the animal. Birds found dead were not included in this category. To assess the rate, the registers of the last 12 months were considered, and a rate was calculated in this way: [culled birds/(culled birds + mortality)] × 100. One hundred points were given when this percentage was equal to or higher than 70%, and 0 points were given when this percentage was lower than 30% (Table 3). In relation to the procedures for emergency killing (10, 11), these were asked of the farmer and, when possible, assessed during the visit. A score of 0 was given if the protocols were correctly drafted and included any of the following systems accurately described: concussion followed by exsanguination, concussion followed by beheading, concussion followed by cervical dislocation, killing with gas, captive bolt, electrical systems, or lethal injection. If the protocols were not correct, then the score was 1. If during the visit an animal was found that needed to be euthanised, it was requested by the farmer for execution and assessed. If the animal was showing signs of consciousness, the score was 2. A score of 0 did not give any points. A score of 1 was resting 55 points to the whole score of the criterion, and if the score was 2, 100 points were resting to the whole score of the criterion.

### 2.4 Appropriate behaviour

The appropriate behaviour principle was assessed by a combination of four criteria: social behaviour (15%), other behaviours (40%), human–animal relationship (30%), and positive emotional state (15%, Table 4).

The social behaviour criterion was assessed using one ABI, which was negative social behaviour (100%). The negative social behaviour (10) was assessed in a total of 10 groups of birds randomly selected at different locations within the farm. Usually, the same birds were used to assess coughing and sneezing. The duration of observation for each group was set at 2 min, during which negative social behaviours were recorded. The authors interpreted the presence of mounts (one quail over another one) as part of a dominance behaviour rather than a reproductive behaviour. Feather pecking and pecking in the area of the cloaca were also observed. Only agonistic behaviour between life birds was considered, and not when any of these behaviours was addressed to a dead animal. Any event of negative social behaviour was recorded as a score of 2 for the specific group of observation, and 100 points were obtained when in none of the groups was observed any event. On the contrary, 0 points were obtained when an event was observed in more than 5 different groups (or periods of observation of 2 min).

The other behaviours criterion was assessed using two ABIs and two RBIs, being: presence and use of the enrichment material (12), including birds digging and interacting with the litter and grooming (30%), plumage damage (30%), outdoor access (30%), and solar light (10%). The first measure assessed the presence of enrichment material as well as the use of these enrichments or a preening behaviour. This parameter was assessed in all the birds observed during the assessment, with a particular focus on those utilised for thermoregulation. Additionally, two additional occasions were conducted, with a minimum of 100 birds being observed each time. These observations included dust bathing, preening, foraging or pecking or scratching the litter anywhere on the farm, and interactions with enrichment material. This last point involved a wide range of behaviours, such as sitting on a bale of straw or hay, a platform or perch, sitting near a bush in outdoor areas, or pecking objects arranged for this purpose. When it was estimated that more than 10% of the birds were performing these activities, a score of 0 was considered. A score of 1 was considered if the percentage was only 10%, a score of 2 if a maximum of 10 individuals were seen performing these activities, and a score of 3 if none of the birds were observed performing any of these behaviours. If the facilities did not provide any kind of enrichment material, a score of 3 would be included. It was deemed enrichment material to include any piece of rope, bales of hay or straw, dust bath areas or stones for pecking. It was also considered an enrichment material if there were dispensers of fibre or grain that required the intervention of the animal to get the food. One hundred points were given if a score of 0 was given to the farm and 0 points when the score was 3 (Table 4). The damage to plumage (12) was assessed in a total of 250 quails sampled in 10 different locations within the farm. It was considered only the dorsal part of the animal, from the end of the back of the head to the beginning of the tail, without considering the wings or the area below them. If all the feathers were present (with the exception of a single feather), the score was 0. If there was a general lack of feathers (moderate wear) but without featherless areas, or if there was one featherless area smaller than 4 cm, the score was 1. If there were any featherless areas larger than 4 cm, the score was 2. The score ranged from less than 0.5% of the birds with a score of 1 and 0% with a score of 2 (100 points) to 30% with a score of 1 or 15% with a score of 2 (o points; Table 4). The presence of an outdoor (free-range) covered area where the birds can access during daylight and with adequate space for at least 80% of the total population of quails was considered (12). One hundred points were given if the outdoor area was present and covered by either artificial or natural means, covering 40% of the surface. If the cover on the outdoor area was less than 40% but was more than 20%, then 55 points were given. If under 20% of the outdoor area was covered, then 0 points were given. The presence of a winter garden or outdoor access that is accessible to the birds at some point during the day gave 40 points. In all the places where the birds were assessed, solar light was assessed as a general measure. A score of 0 was given if all the birds had access to sunlight for at least some hours per day, a score of 1 if at least 50% of the birds had access to sunlight, and a score of 2 if less than 50% of the birds had access to sunlight (Table 4).

The good human–animal relationship criterion was assessed through one ABI and one RBI, being avoidance distance test (50%) and staff training (50%). The avoidance distance test was assessed in a minimum of 10 groups of birds, and it was based on the Welfare Quality^®^ Broiler Protocol (12). This test was performed as follows: the observer slowly approached a group of birds and, before doing anything else (while standing and making as few movements as possible), counted how many birds there were around her/him. Then she/he crouched down, waited 10 s, and observed around her/him the percentage of birds that remained less than one metre away, evaluating the percentage of birds standing still or sitting in good condition (score 0), the percentage of birds moving around (score 1), and percentage of birds fleeing (score 2). The score varied from less than 10% of the birds with a score of 1 or 2 (100 points) to 70% of the birds with a score of 1 or 2 (0 points, Table 4). Training of personnel considered three levels (10, 11): all personnel in the farm in contact with the birds were trained in animal welfare, score 0 (100 points); at least one person was trained in animal welfare, score 1 (55 points); and none of the persons were trained in animal welfare, score 2 (0 points). Certificates of attendance for any training must be presented.

The positive emotional state criterion was assessed through one ABI, which was a novel object test (100%). The Novel object test was assessed in a minimum of 10 groups of birds and was based on the test employed for laying hens in Welfare Quality^®^ (12). To conduct the test, a stick with a length of 20 cm and a diameter of 3 cm was used with a combination of at least three colours (a novel object). The assessor waited for 30 s in front of the birds prior to starting each test before removing the novel object from the ground. After this period, the object was left on the ground, and the observer moved one and a half metres away. From then on, it counted the number of quails that were less than one quail away from the novel object every 10 s, up to a maximum time of 1 min. A score of 0 was given if within the first 30 s, two or more birds approached the novel object, or if within the 60 s, four or more birds approached the novel object. A score of 1 was given if within the first 30 s, one animal approached the novel object or if within 60 s, three birds approached the novel object. A score of 2 was any other situation. The score ranged from 5 points with a score of 0 and a maximum of two points with a score of 2 (100 points) to more than 8 points with a score of 2 (0 points, Table 4).

### 2.5 Overall assessment

After observing the quails and the facilities at 10 different locations per farm, an overall assessment was carried out. This protocol was implemented by adapting the calculation model and the categorisation provided by the Welfare Quality^®^ protocol. In this regard, in the present protocol, a fixed score system was employed, assigning a specific value to each measure, criterion, and principle. However, in Welfare Quality^®^, the weight of a measure or criterion can vary according to its score, and the final score is not obtained by using an average, as we did. However, the categories used were identical, with the same thresholds. Thus, the final score for each farm was the result of the combination of the four principles according to the importance and burden of the indicators, criteria, and principles (Figure 1), as follows: 15% depends on Good feeding, 30% on Good housing, 30% on Good health, and 25% on Appropriate behaviour. With an overall score ranging from 0 to 100 points, four welfare categories were obtained, as follows: “not acceptable” (0–19) when the welfare of birds in the farm was low and considered unacceptable; “acceptable” (20–54) when the welfare of birds was above or meets minimal requirements; “enhanced” (55–79) when the welfare of birds was good; and “excellent” (80–100) when the welfare of the birds was of the highest level.

### 2.6 Statistical analysis

The welfare score calculation was based on the calculation model proposed in the Welfare Quality^®^ protocol, with some modifications. The overall score for each indicator, criterion, and principle ranged from 0 to 100, and each farm was classified into four categories. Descriptive analyses were employed to present the collected measures by utilising the mean and standard deviation (mean ± SD).

## 3 Results

### 3.1 Good feeding

According to the criterion of Absence of prolonged hunger, as shaped by the indicators of Body condition, Birds per feeder and Cleanliness of feeder, a total of 13 meat quail farms assessed were classified as “enhanced” and one as “excellent” (73.4 ± 5.63). For the indicators of Body condition and Cleanliness of feeder, all farms were classified as “excellent”, but outlined insufficient feeder space per animal (0.3 ± 0.04 cm/animal) being categorised as “not acceptable” (*n* = 6), “enhanced” (*n* = 1), and “acceptable” (*n* = 7). Similarly, the Absence of prolonged thirst score (50.7 ± 12.54) was calculated through “birds per drinker”, “functioning of drinkers”, and “cleanliness of drinkers” scores. Among the farms with the lowest score for this criterion (“acceptable”, *n* = 7), four of them showed at the same time an insufficient space of drinkers (40 ± 7 birds per drinker), by which they were classified as “not acceptable” for this indicator. On the contrary, all farms evaluated showed excellent cleanliness of the drinkers as well as functioning properly; two of them were classified as “excellent”.

**Figure 2:**
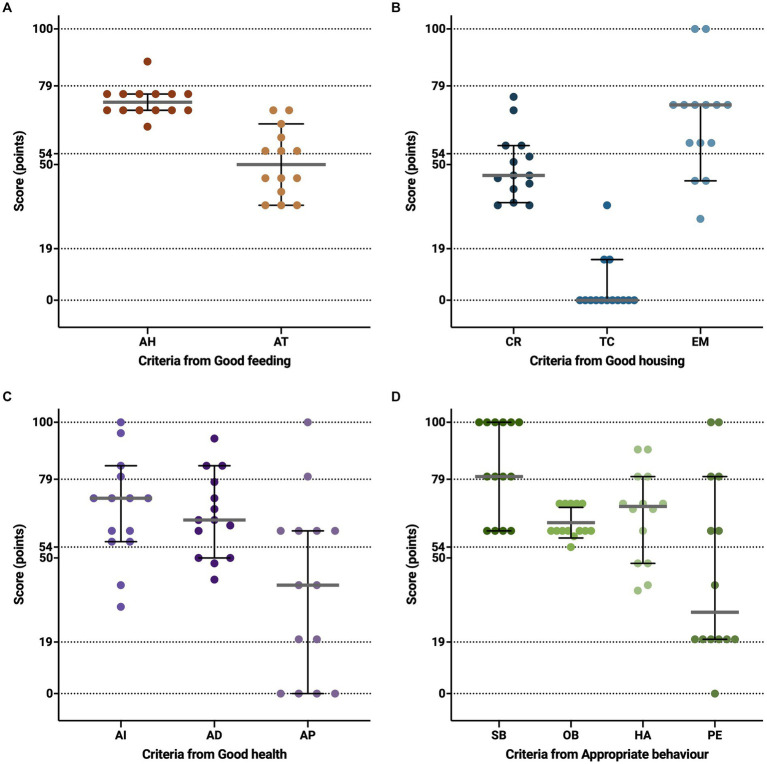
Dot plots representing the score achieved on the 13 criteria utilised for the assessment of animal welfare in 14 meat quail farms. In **(A)**, the criteria for Good Feeding: Absence of prolonged hunger (AH) and the Absence of prolonged thirst (AT). In **(B)**, the criteria for Good Housing: Comfort around resting (CR), Thermal comfort (TC), and Ease of movement (EM). In **(C)**, the criteria for Good Health: Absence of injuries (AI), Absence of diseases (AD), and Absence of pain induced by management (AP). In **(D)**, the criteria for Appropriate Behaviour: Social behaviour (SB), Other behaviour (OB), Human–animal relationship (HA), and Positive emotional state (PE). In the criteria, the grey line represents the median and the black line represents the 95% confidence interval (CI). The dashed lines represent the range of the category where farms are classified: “not acceptable”, from 0 to 19; “acceptable”, from 20 to 54; “enhanced”, from 55 to 79; and z’excellent’, from 80 to 100.

Thus, the Good Feeding Principle score ranged from 54 to 75 (63.2 ± 6.53), with most farms classified as “enhanced” (*n* = 12) and two farms as “acceptable”. Farms scores for the criteria included in this principle are shown in Figure 2A.

### 3.2 Good housing

This principle is assessed by means of the combination of three criteria: Comfort around resting, Thermal comfort, and Ease of movement.

The comfort around resting score (49.3 ± 12.28) is computed considering the Plumage cleanliness, the percentage of Wet and sited birds, the Litter quality, the level of dust, and the Light quality scores. In relation to plumage, 13 farms housed birds with the presence of dirt, but only one of them had problems with the presence of birds with a wet plumage. Evaluating the Sited birds indicator, two farms were considered as “excellent” most of the farms were assessed “acceptable” (*n* = 9) and, to a lesser extent, “enhanced” (*n* = 3). The Litter quality was classified as “excellent” in the half of farms assessed (*n* = 7) while other farms were categorised as “enhanced” (*n* = 4) and “acceptable” (*n* = 3). The Dust sheet test was “acceptable” in most farms (*n* = 12) but “not acceptable” in two. Because no farm was provided 8 h at least of darkness, the Light quality indicator was scored as 0 in all farms and was classified as “not acceptable” (*n* = 14).

The thermal comfort score is calculated considering the percentage of Panting or shivering birds and the Temperature and humidity recordings. Due to that, the temperature and humidity were not recorded in the majority of the farms assessed (*n* = 11), and taking the points discounted into consideration, only one farm exceeded the 20 points required to be considered as “acceptable”, this criterion showing the lowest score (4.6 ± 10.28) of welfare protocol on quail.

Finally, the ease of movement considers two indicators: the capability of birds’ movement and the Stocking density. The capability of birds’ movement was observed in all farms; hence, all farms were classified as “excellent” for this indicator (*n* = 14). In terms of stocking density, on average, each quail had an area of 114 cm^2^ (89–147 cm^2^/quail). Thus, approximately 80% of farms were classified as “enhanced” (*n* = 6) and “acceptable” (*n* = 5), two farms as “excellent” and one had “not acceptable” categorisation. Finally, the Ease of movement criterion score (66.0 ± 19.58) was classified as “enhanced” in nine farms and in least a minority as “acceptable” (*n* = 3) and “excellent” (*n* = 2) with scores ranging from 30 to 100. Farms scores for the criteria that compound the Good housing principle are shown in Figure 2B.

Among all the principles, the Good Housing Principle scored the lowest (49.4 ± 9.36), ranging from 28 to 61. The farms were classified as “enhanced” (*n* = 4) and “acceptable” (*n* = 10).

### 3.3 Good health

Among the three criteria, within the principle of Good health, the criterion of Absence of injuries was considered, consisting of the prevalence and severity of Wounds on the body, Lameness, Footpad dermatitis and Hock burn, as well as the Risk of injuries. In terms of injuries to the body, in half of the farms assessed, the prevalence was 0%, and in three of the farms, the prevalence was less than 0.5%. Hence, the score of Wounds on the body (80–100 points) was classified as “excellent” in 10 farms; the remaining farms were considered as “acceptable” (*n* = 3) and “enhanced” (*n* = 1). Only two farms were classified as “excellent” for the indicator Lameness, showing prevalence <1 and 0% for moderate and severe lameness, respectively. Thus, farms were mostly classified as “acceptable” (*n* = 8) and “enhanced” (*n* = 3) and, marginally as “not acceptable”. Footpad dermatitis was observed in a moderate state in only two farms, showing a prevalence of ˃ 2.4% on all farms categorised as “excellent”. The Toe damage was found in less than 8% of birds in eight farms that were classified as “excellent” and above 20% of birds in two farms being “not acceptable” for this criterion. The Hock burn was found by the presence of birds with dermatitis on the hock joins; farms with a prevalence <1% were classified as “excellent” (*n* = 5), and prevalences above 1% were classified as “enhanced” (*n* = 2) and “acceptable” (*n* = 7). Finally, none of the farms assessed had a Risk of injuries due to the surroundings. Since no points were deducted in the criterion of the absence of injuries on any farm, the final score (68.0 ± 19.15) resulted in an overall criterion classification of “excellent” (*n* = 4), “enhanced” (*n* = 8), and “acceptable” (*n* = 2).

The absence of disease score is calculated by considering Mortality and the prevalence of Coughing/sneezing birds, Nasal or ocular discharges, and Diarrhoea. In farms, the prevalence of accumulated mortality at 7d ranged from 0.32 to 5.7%, and total flock mortality ranged from 0.5 to 10.5%. Only one farm was rated “excellent”, demonstrating percentages of mortality <2% for all productive cycles and < 1% within the first week. Contrary, a prevalence above 10% for flock mortality and 5% in the first week positioned three farms as “not acceptable”. For the indicators of coughing/sneezing and eye/nasal discharges, all farms obtained scores included in the “excellent” category. The Diarrhoea indicator had a 0.5–0% prevalence in eight farms valued as “excellent”; the rest were classified as “enhanced” (*n* = 3) and “acceptable” (*n* = 3). The overall result for this was scored with 65.7 ± 15.41.

Finally, for the criterion of Absence of pain induced by management were considered two indicators. Culling on the farm was calculated in relation to the total of quail found dead on the barn floor. Four farms were rated as “enhanced” and four as “acceptable” for having records of culling birds between 59 and 30% with respect to the total number of dead quail, and only two as “excellent” with records above 60%. The emergency killing method was appropriate in all farms, so no points were deducted from the final criterion score (38.6 ± 32.78).

Based on the outcomes of the three criteria that conform to the Good health principle (Figure 2C), farms were classified as “acceptable” (*n* = 5), “enhanced” (*n* = 8), and “excellent” (*n* = 1), with a total score ranging from 45 to 85 (62.6 ± 12.68).

### 3.4 Appropriate behaviour

The Appropriate behaviour principle is assessed by means of the combination of four criteria: Social behaviour, Other behaviours, Human–animal relationship, and Positive emotional state, whose results are shown in Figure 2D.

In relation to the criterion of Social behaviour, this was conformed to one indicator demonstrating that events of negative social behaviour were not found in the majority of farms assessed (*n* = 10) being qualified as “excellent”. Nevertheless, feather pecking was observed in four farms rated as “enhanced”. Consequently, this criterion was scored with 82.9 ± 17.29. The criterion on the expression of other behaviours was assessed considering four different indicators: the Presence and use of enrichment material, digging or playing with sand and grooming, prevalence and severity of Plumage damage, presence or absence of Solar light, and possibility to access an outdoor range or Outdoor access. For indicators of Presence and use of enrichment material and plumage damage, quail with these assumptions were observed in all farms assessed. Thus, all farms were classified as “excellent” in terms of the presence and use of enrichment material, foraging scratching, and preening. On the other hand, although most farms had high scores for the Plumage damage indicator, only one was classified as “enhanced” for housing quails with moderate and severe plumage damage. Instead, all farms were rated as “not acceptable” for not having access to outdoor areas. For the Solar light indicator, farms were categorised as “excellent” (*n* = 6) or “not acceptable” (*n* = 8) depending on whether or not the birds had access to sunlight. Considering the four indicators for expression of the Other behaviours criterion, farms were globally rated as “enhanced” (*n* = 13) and “acceptable” (*n* = 1), showing a score of 63.0 ± 5.64. The human–animal relationship criterion includes the assessment of two indicators: the human approach test and welfare Staff training. The human approach test resulted in an average of 61.6% (35.0–83.1%) of birds standing or sitting, 9.9% (1.5–18.4%) of birds moving around, and 28.5% (5.2–56.5%) of birds running away, leading to half of the farms being classified as “acceptable” and the rest as “enhanced” (*n* = 2) and “excellent” (*n* = 5). In eight farms, staff in contact with birds were trained in specific courses on animal welfare and were rated as “excellent”. On five farms, not all staff, but at least one person, was trained in animal welfare, qualified as “enhanced”, and one farm obtained the “not acceptable” qualification. The combination of the two indicators resulted in an overall criterion classification of “excellent” (*n* = 4), “enhanced” (*n* = 6), and “acceptable” (*n* = 4), with scores ranging from 38 to 90 (65.7 ± 16.97). Finally, the Positive emotional state criterion was assessed by only one indicator. Thus, the outcomes of the Novel object test allowed to classify farms in the criteria as “excellent” (*n* = 4), “enhanced” (*n* = 2), “acceptable” (*n* = 7), and “not acceptable” (*n* = 1) with scores of 45.7 ± 33.68.

Within the principle of appropriate behaviour, a score of 64.1 ± 9.72 was allowed, classifying 12 farms as “enhanced” and two as “acceptable”.

### 3.5 Overall assessment

Considering the global score of all the farms (59.1 ± 5.21), the majority of farms were defined as “enhanced” (*n* = 11), ranging from 51 to 66 scores, and three as “acceptable” with a rating between 51 and 54 points. No farm was classified as “excellent” or “not acceptable”. In terms of animal welfare certification, the three farms, including those in the “acceptable” category, would not achieve the minimum for its certification (that should be an enhanced level). The other farms (*n* = 11) would be certified, but in most cases, they were too close to the threshold of 55 points, so some improvements should be suggested.

## 4 Discussion

In this study, 14 quail farms were assessed during their first year of integration in an animal welfare certification scheme. As a result, the findings may be subject to two potential biases. First, the farmers are aware of the assessment protocol in advance and enter the programme voluntarily, suggesting that only those who are confident in their farms’ ability to meet the requirements would apply. Second, to be approved, a score of “enhanced” is necessary for the overall assessment, so it is only expected that farms with very poor self-assessments would have global scores below 55 points. Given these factors, the study examined the variability between the assessed farms and the feasibility of the indicators used, paying special attention to the validity and reliability of animal-based measures and the overall assessment of meat quail welfare protocol.

### 4.1 Good feeding

The quail breeding species is the one used for both meat and egg production (5). However, a heavier line of this Japanese subspecies is selected for meat production due to its pattern of body and muscle growth (13). Within the Good Feeding Principle, the ABI Body condition was used to ascertain the percentage of lean birds, paying special attention to the development of the muscle around the keel, of particular importance, namely supracoracoideus and pectoral muscles (14). Using this indicator, no differences were found between farms, showing all of them had excellent values in relation to the body condition. This may be because all the birds on the farms evaluated were from the heavy line of the Japanese subspecies, characterised by a high development of the pectoralis muscle (15) along with the production system where birds are usually fed *ad libitum*. Although it is described that females are heavier than males, no data related to the sex of the birds was collected in the present study. This is because meat quails are usually slaughtered before reaching sexual maturity (16), and there is no marked sexual dimorphism (17) with respect to weight.

Other indicators included in this principle did not reach the minimum required by the protocol. This is the case of Birds per feeder and Birds per drinker. This is because the legislation (Council Directive 2007/43/EC) is only asking that the farms must have enough feeders and drinking points, properly distributed and easily accessible, to ensure maximum availability for all birds, without specific values. On the other hand, the European Food Safety Authority (EFSA) (5) presented a range between 30 and 52 quail per drinker. With these values, the farms would be assessed as “not acceptable” with this protocol. The guide published by Aviagen (18), focussing on chicken production, recommends installing one cup drinker per 20–30 birds and a space of 5 cm per bird for pan feeders, which would be categorised as acceptable in this protocol. In the future, it will be necessary to address a specific study to ascertain if the protocol is too severe at this point and need to apply other ratios to this measure.

### 4.2 Good housing

The results obtained from Good Housing showed that it was the principal with the lowest score. Analysing the three criteria that compound this principle, in all farms Comfort around resting had a positive assessment, although none farm achieved the “excellent” category. Although there are no published studies on plumage cleanliness in quails, this measure has been addressed in other poultry species (19–22), and it is considered important because feather cleanliness is involved in thermoregulation, protection against moisture and dirty, and skin infections (23), and with a comfort behaviour (5). Therefore, the data obtained in these farms and others assessed with the protocol could be used as a basis to know the state of the art of this important indicator. Although in the EFSA review (5) the term “wet” refers more to soil conditions than to the birds themselves, another important point to consider in the comfort of birds is to have dry feathers. In this respect, the percentage of wet birds in the protocol was low, with more than 90% of farms having an “excellent” in this indicator. However, it is clear that in most cases, wet birds are related to other indicators such as plumage cleanliness and litter quality (5). In this context, the Litter quality showed an “excellent” score in half of the assessed farms, all of them with the same qualification for the indicator wet birds. However, the other half had a lower score in litter quality that was not translated to more wet birds.

It is widely known that rest and sleep are important stationary behaviours for the welfare of birds (24); however, only two farms obtained the “excellent” qualification for the indicator Sited birds. To improve it, it is suggested to provide structures to allow the quails to rest under cover (EFSA) (5). Attending to other indicators under the criterion of Comfort around resting, the animal welfare EU legislation (Council Directive 98/58/EC) includes the assessment of dust levels so that they are kept within limits and which are not harmful to birds located on the farm. There are different evaluation methods for assessing dust levels, and despite the dust sheet test being subjective, it has been recognised to be a valid indicator for measuring dust levels in poultry barns (25). Notably, it is the reference method used by Welfare Quality^®^ for dust levels in poultry (12). In the present study, all farms assessed obtained a “poor score”. This could be explained by the physiological behaviour of quail to perform dust baths, being considered a positive aspect of the principle of Appropriate behaviour but conditioning other environmental aspects that can affect quail welfare. At last, the Light quality indicator was scored with a 0 (the lower score) in all farms. The maintenance of light programs with the appropriate duration, distribution and intensity has an impact on the productive parameters of poultry species, including meat quail (26) and these can affect the welfare and behaviour of birds (27). The light programs for broiler farms establish a period of 8 h of light and 8 h of darkness, which can be supplemented with artificial lighting up to a maximum of 16 h of light. Gharaoghlan et al. (28) studied different lighting programmes concluding that 8 h of light would have beneficial effects for meat quail. Despite this, it is common for quail meat production to find long light programs up to 16 h. In the case of the 14 farms evaluated, all of them had established lighting that did not conform to these recommendations, and for this reason, they were rated with 0 points.

The EFSA (5) displayed that the thermal comfort of quails, specifically in laying quail, is approximately 23°C, so birds can show signs of cold or heat stress such as panting, shivering, huddling, and spreading of the wings in temperatures clearly below or above this number. Above that, the criterion proposed by this protocol called “thermal comfort” allowed us to evaluate it in the birds and the housing, obtaining very low scores in all the farms assessed. This was not always because of the presence of a high percentage of birds showing thermal stress, but in most cases because of the lack of records on the environmental condition records. This is a clear and easy point to improve for the farmers in the future. Closely related to the above, another environmental stressor included is the criterion “ease of movement” is the space allowance (stocking density). This is a critical point because it affects the presence of natural behaviours, such as performing short flights, or dust bathing, and affects ventilation and the availability of arrival resources, such as food and water (29). The results showed that all farms had adequate height and horizontal space, and this allowed 100% of the birds to be able to run, jump, and perform short flights. For the “stocking density”, EFSA (5) recommends, based on current practices, a stocking density of approximately 125 birds/cm^2^. This ratio is met by eight of the 14 farms assessed in the present protocol.

### 4.3 Good health

Under the criterion of Absence of injuries, it is assessed the presence of skin damage and wounds in quails. EFSA (5) defines wounds as all lesions to the skin ranging from minor superficial punctiform spots or scratches to large open wounds and interprets this as a probable consequence of restriction of movement. In general, the results obtained in the present study were very encouraging because, even though there are no studies on this subject in quails, 10 farms obtained a score of excellent. Similarly, there are no published studies in quail on the relationship of locomotor disorders with the rest of the indicators included in this criterion (Lameness, Footpad dermatitis, Toe damage, and Hock burn). However, in other species, the indicators of this criterion have been related to other indicators included in the protocol. For instance, in broiler chickens, the presence of severe lameness has been linked to litter and air quality (30), similar to foot pad dermatitis and hock burns can be affected by litter quality (31). Regular upkeep of facilities and prompt replacement of feeders and drinkers before they become worn out can prevent quails from sustaining injuries. A low score on this ABI could be used as a sign of poor housing conditions. However, in the present study, 100% of the farms assessed were in good status.

In relation to the Absence of diseases criterion, the most important to consider is mortality, which represents the failure of an animal in coping with environmental challenges. The Mortality indicator in the present protocol is evaluated by assessing total and first week mortality. In poultry meat production, mortality during the first 7 days of life is postulated as an important indicator of production (32) that can be influenced by factors such as genetic line, infectious agents, and environmental and breeding conditions (33). In this context, El Sabry et al. (29) suggested that farms with reduced space could have an increase in the mortality rate. The results obtained from the implementation of this protocol in the 14 farms revealed in three of them high mortality percentages, unrelated to stocking density, that were rated as “not acceptable” for this criterion.

Even though for the indicators of coughing, nasal and ocular discharge, and diarrhoea, the EFSA (5) did not report any related sources, there are publications that show these types of symptoms and the susceptibility of quail to certain bacterial, viral, mycotic, and parasitic diseases (34, 35). In this case, the results obtained are not poor but could be improved, with more than 70% of farms scoring above 50 points for the Absence of diseases.

The last criterion, Absence of pain induced by management, showed that approximately 30% of farms are not implementing the culling of birds before they die of disease or injury. In this regard, this is a critical point for improvement, as birds should be euthanised when signs of not recovering and suffering are identified. Furthermore, proper recording of these animals and the reason for euthanasia are crucial to improving the welfare of the entire system in the future.

### 4.4 Appropriate behaviour

In the commercial husbandry of meat quails, indoor floor systems are used, which means that males and females live together from their entry into production until slaughter. The establishment of hierarchies in birds results in social interactions that are commonly presented as negative affective experiences (5). In the present protocol, the indicator Negative social behaviour, based on the presence of agonistic behaviour in birds, showed a good result, with approximately 70% of farms evaluated with an “excellent”, but with possibilities of improvement in the rest of the farms. In the criterion Other behaviour, four indicators were chosen. As mentioned above, quails were housed in an indoor floor system with no access to the outdoors, so low scores in the evaluations of two (Solar light and Outdoor access) of the four indicators that compound the Other behaviours criterion are to be expected. The other two indicators, Plumage damage and Presence and use of enrichment material (both ABIs), were positively scored. In quail, the aggressive pecking is indicative of welfare problems and is manifested as plumage damage (29). As regards the indicator Presence and use of enrichment material, EFSA (5) revealed that environmental enrichment is usually not provided, although this is not the case for the farms assessed in the present study.

Regarding the human–animal relationship criterion, the farms obtained a good score because the regulation asks for training on animal welfare, so in this specific case, the protocol is confirming that the regulation is correctly applied in the farms assessed. Finally, a Novel object test is used to assess the global emotional state of the birds, especially the presence of fear (36, 37). Although all the farms had similar management and used the same breed of birds, differences between farms were detected in the percentage of animals approaching a novel object.

### 4.5 Overall assessment

As mentioned above, the voluntarily included farms were evaluated using the animal welfare protocol for meat quails developed in the present study, so it would be obvious that none of them had a very low score. The minimum overall score required to be certified by this protocol was 55 points; therefore, only those categorised as “enhanced” or “excellent” would be expected. However, three farms did not achieve the minimum score, although they were very close (51–54 points). Predictably, the review and improvement of those evaluated critical indicators could improve the overall score and, therefore, achieve the higher category. The results obtained, shown in Figure 2, demonstrated that among all the principles, the Good housing principle showed the most deficient scores; specifically, the Thermal comfort criterion presented very low scores in all the farms evaluated across the board. This was because a total of 11 farms did not have records of environmental conditions (temperature and humidity). Within the principle of Good health, the criterion of Absence of pain induced by management, evaluated by the culling indicator, highlights the need to evaluate individually those quails with severe health problems in order to treat and/or euthanise them to prevent suffering and mortality on the farm. In the Appropriate behaviour principle, the criterion Positive emotional state assessed by the Novel object test showed lower scores with wide variability between farms. Of all the farms evaluated, none was rated as “excellent” therefore, in all cases, there are opportunities for improvement, and the protocol that is proposed in this study can be used as well as a tool for identifying gaps and planning future investments. However, a future version of the protocol could attempt to add more variability to the first principle and penalize the final value more if the last one has such low scores. The results show how, for years, meat quail producers have been very focused on feeding needs and very few on behavioural needs. On the other hand, although all the farms had similar management, came from some country and used the same breed, the protocol seems to be useful for identifying differences in individual indicators and the overall score, being a useful tool for benchmarking. In any case, a future protocol should consider new indicators, e.g., ammonia concentration within the good housing principle and a system to penalize more the farm when certain measures, such as lameness, score too low due to a high prevalence of a problem.

It is clear that this protocol can be used in different ways. For example, other researchers can test the individual measures used in this protocol in contexts other than the Spanish farms where they have been tested, even in real or laboratory conditions. Similarly, specific sections, such as the Good Health principle, can be selected for studies that address only the health of these animals. On the other hand, farmers can use the tool as a whole to carry out a self-assessment of their own farm to compare it with those already assessed in this protocol or even with others that may appear in the future and, finally, it can be used as an animal welfare certification tool on the condition that continuous improvement is applied. This means that the scoring system and thresholds will have to be adapted to produce measurable improvements in terms of animal welfare. In fact, since 2022, when these farms were assessed, most of them have implemented strategies to increase the score in the measures identified with the lower scores, so the system, when used properly, can also be very useful not only to do an assessment of the situation of a farm but to plan future improvements that may affect the most critical points that impact on animal welfare.

## 5 Conclusion

In general, most of the farms received a good overall score, with the highest score being 66 points. However, none of the farms earned an “excellent” rating, and three farms scored below the required 55 points. The Good Feeding Principle received the highest score, achieving an “enhanced” rating in all farms, while the Good housing principle received the lowest score, ranging from 28 to 61 points out of 100. The protocol demonstrates a significant level of diversity at the indicator level, making it a valuable tool for benchmarking and enhancing the welfare of quails, particularly in areas that have been identified as needing improvement. The main welfare problems detected in all farms were the lack of temperature and humidity records, the lighting pattern and light quality, and the absence of an outdoor range or provision of outdoor access. Additionally, some farms had excessive numbers of birds per feeder, improperly functioning drinkers, poor litter quality, and a high prevalence of birds with dirty plumage and lameness. Culling of animals should be improved to prevent suffering, and, in general, quail producers should focus on behavioural needs as they have traditionally done with feeding needs.

## Data Availability

The original contributions presented in the study are included in the article/Supplementary material, further inquiries can be directed to the corresponding author.
