# Supplementary material for: Animal welfare assessment protocol for quails reared for meat production
Source: Front Vet Sci. 2024 Sep 30;11:1452109. doi: 10.3389/fvets.2024.1452109 (PMC11471728; doi:10.3389/fvets.2024.1452109)
Supplement: Supplementary file 1 [file Data_Sheet_1.pdf]

Supplementary Figure 1. Photografic examples of the measures: feather pecking, food pad dermatitis, toe damage and hock burn,

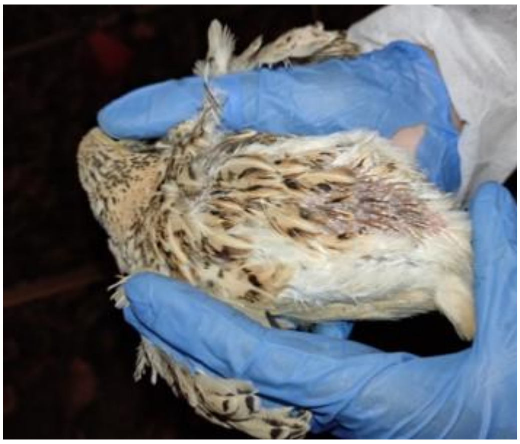

1A) Photografic example of feather pecking (score 1)

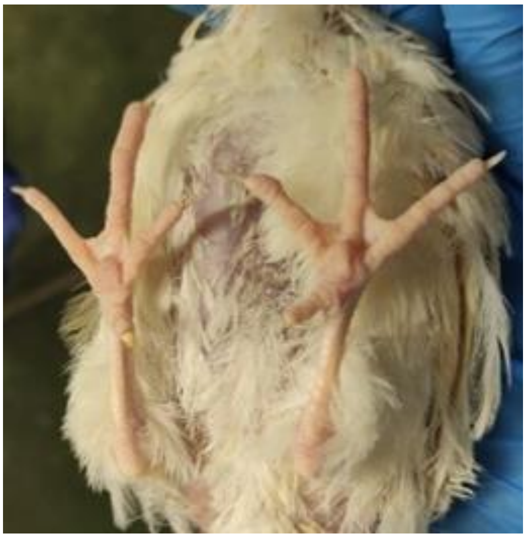

1B) Photografic examples of Foot Pad Dermatitis (score 0) and Toe Damage (score 0)

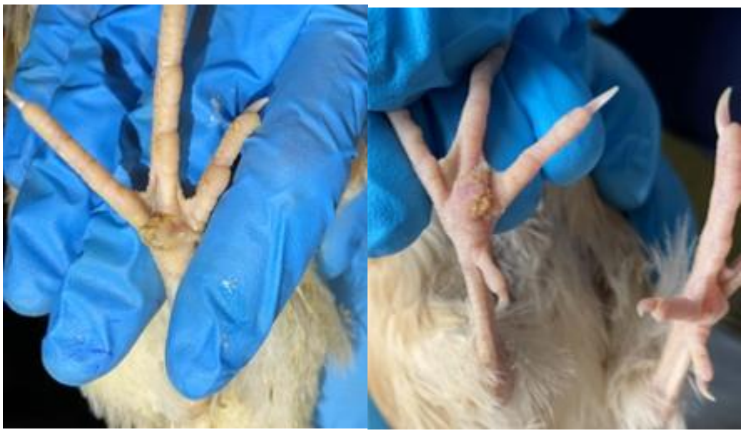

1C) Photografic examples of Foot Pad Dermatitis (score 1)

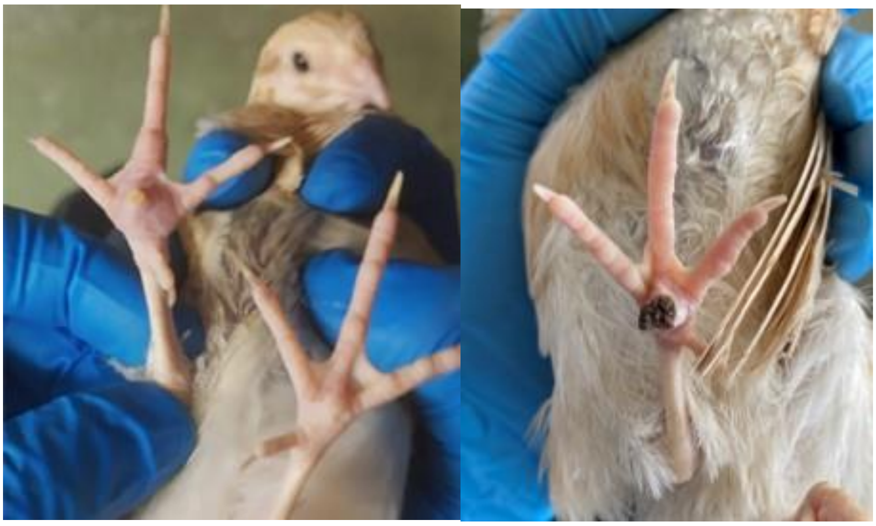

1D) Photografic examples of Foot Pad Dermatitis (score 2)

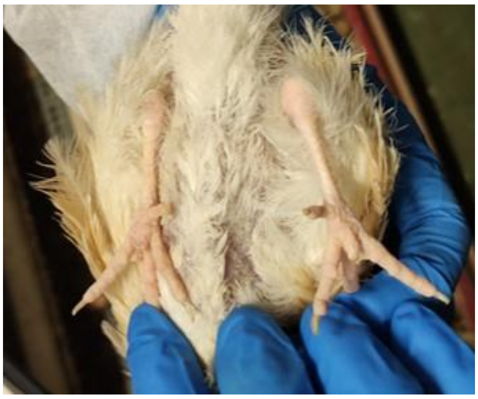

1E) Photografic example of Hock Burn (score 0)

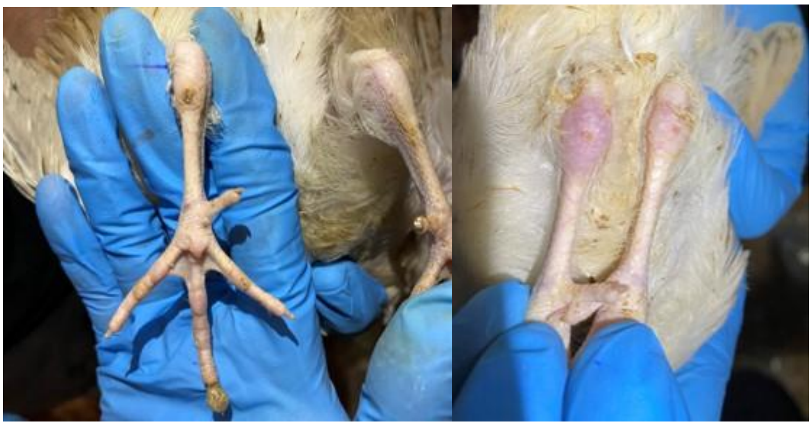

1F) Photografic examples of Hock Burn (score 2)

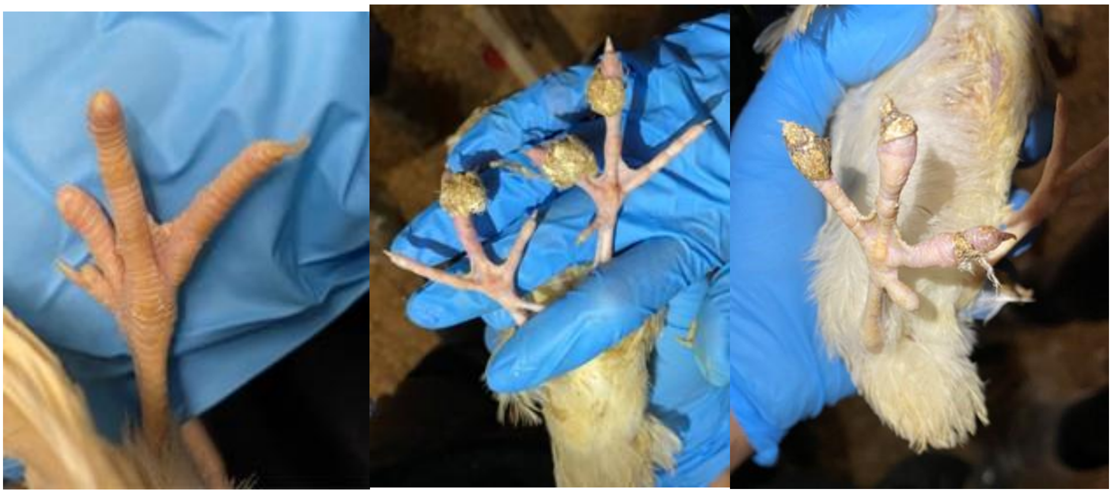

Photografic examples of Toe Damage (score 2)
